# Next-Day HIV Viral Load Test Result and Linkage to Care Among Persons Living With or at Risk of HIV: A Randomized Clinical Trial

**DOI:** 10.1001/jamanetworkopen.2025.48380

**Published:** 2025-12-16

**Authors:** Matthew M. Hamill, M. Harris Bayan, Alec Boudreau, Nisha Ramdeep, Richard Rothman, Susan H. Eshleman, Tanique Bennett, Thelio Sewell, Zoe O. Demko, Agha Mirza, Travis J. Smalls, Nyah Johnson, Benji Riggan, Elizabeth Nielsen, Robin J. MacGowan, Nathalie Gonzalez-Jimenez, Pollyanna R. Chavez, Kevin P. Delaney, Yu-Hsiang Hsieh, Yukari C. Manabe

**Affiliations:** 1 Division of Infectious Diseases, Johns Hopkins School of Medicine, Baltimore, Maryland; 2 Department of Emergency Medicine, Johns Hopkins School of Medicine, Baltimore, Maryland; 3 Department of Pathology, Johns Hopkins School of Medicine, Baltimore, Maryland; 4 Centers for Disease Control and Prevention, Atlanta, Georgia

## Abstract

**Importance:**

HIV viral load (VL) testing is essential for monitoring responses to antiretroviral therapy (ART) among people with HIV (PWH) and prior to the initiation of HIV preexposure prophylaxis (PrEP). In the US, the potential benefit of implementing HIV VL testing in these scenarios on linkage to care (LTC) has not been evaluated.

**Objectives:**

To investigate whether providing laboratory-based HIV VL test results changed LTC rates or time to linkage for ART or PrEP across 12 weeks.

**Design, Setting, and Participants:**

This randomized clinical trial was conducted from August 18, 2021, to February 2, 2023, with 12 weeks of follow-up. Participants were a convenience sample of adults with risk factors for HIV acquisition or PWH not taking daily ART, all of whom were recruited from an academic center emergency department in Baltimore, Maryland, and via social media advertising.

**Interventions:**

Participants were randomized 1:1 to receive a laboratory-based plasma HIV VL test with next-day results in addition to the standard of care HIV antigen/antibody test result (intervention) or to receive the standard of care HIV antigen/antibody assay alone (control).

**Main Outcomes and Measures:**

The primary outcome was LTC for ART or PrEP within 12 weeks of enrollment. Secondary outcomes included time to LTC and differences in LTC by HIV status. Analyses were conducted using the intention-to-treat population.

**Results:**

Of 1105 potential participants screened, 195 (17.6%) were enrolled (median [IQR] age 36, [27-47] years; 119 [61.0%) male; 112 [57.4%] Black or African American, 51 (26.2%) White, and 32 (16.4%) other race and ethnicity; and 34 [17.4%] PWH). By week 12, 93 participants (47.7%) completed follow-up, and 69 (35.4%) were linked to care (38 of 69 [55.1%] in the intervention group vs 31 of 69 [44.9%] in the control group). Overall, there was no statistically significant difference in LTC between the intervention and control group (hazard ratio, 1.28 [95% CI, 0.80-2.05]; *P* = .31]). In a modified intention-to-treat analysis, time to LTC was significantly less for PWH in the intervention group (log-rank *P* = .03).

**Conclusions and Relevance:**

In this randomized clinical trial assessing the effects of HIV VL test results on LTC, providing a next-day HIV VL test result did not change LTC overall. More data are required to ascertain whether a rapid point-of-care HIV VL test would improve LTC.

**Trial Registration:**

ClinicalTrials.gov Identifier: NCT04793750https://clinicaltrials.gov/study/NCT04793750

## Key Points

**Question:**

Does providing a next-day HIV viral load (VL) test result increase linkage-to-care (LTC) rates among people with HIV or with risk factors for HIV acquisition?

**Findings:**

In this randomized clinical trial that included 195 adults, there was no difference overall in LTC among adults who did (intervention) or did not (control) receive an HIV VL test result.

**Meaning:**

Provision of an HIV VL test result did not improve LTC in persons with or at risk of HIV infection.

## Teaser

This randomized clinical trial investigates whether providing a next-day HIV viral load test result increases linkage-to-care rates among people with HIV or with risk factors for HIV acquisition.

## Introduction

To reach the 95-95-95 goals^1^ and End the HIV Epidemic^2^ by 2030, early detection of undiagnosed HIV, linkage to care (LTC), and rapid initiation or reinitiation of antiretroviral therapy (ART) with viral suppression are required. For persons at risk for HIV, access and continued adherence to HIV preexposure prophylaxis (PrEP) is necessary. The access point to HIV treatment and prevention is HIV testing, to diagnose individuals with infection and identify those who would benefit from prevention interventions. Combined HIV antigen/antibody (Ag/Ab) assays have increased sensitivity compared with older diagnostic tests that only detect HIV antibodies.^3^ However, Ag/Ab tests fail to identify individuals with very early HIV infection, which also has implications for PrEP initiation.^3^ The only US Food and Drug Administration (FDA)–approved self-test is antibody only.^4^

The World Health Organization recommends a treat all approach to HIV to minimize the time from HIV diagnosis to ART initiation^5^; a viral load (VL) test is not required prior to ART initiation. In the US, the Panel on Antiretroviral Guidelines for Adults and Adolescents recommends HIV VL testing before ART initiation but does not mandate that the result be available prior to initiating ART.^6^ In addition, the US FDA has approved VL monitoring after HIV diagnosis.^7^ HIV Ag/Ab testing plus an HIV VL is required for people who have features of acute HIV infection and people who have recently (within 12 weeks) taken oral PrEP and prior to administration of long-acting injectable cabotegravir PrEP.^8^ Access to an HIV VL test result may improve health care professional–patient communication and can inform clinical decision-making. Clinicians may be more likely to initiate PrEP for a patient recently exposed to HIV with a negative VL test result. We hypothesized that rapid provision of a VL test result would reduce decision-making time and increase the likelihood that patients receive their result and are linked to care.

Rapid, or point-of-care (POC), VL tests are not approved by the US FDA for use in the US but are used in other countries^8,9,10^; other POC VL tests are in development.^11^ In the absence of a POC VL test with a result available within a clinical encounter, we investigated whether the use of a central laboratory VL test with a turnaround time (time from sample collection to result being available) of approximately 24 hours would impact linkage to ART for people with HIV (PWH) and to PrEP for individuals with risk factors for HIV acquisition.

## Methods

The methodology and inclusion and exclusion criteria for the Ending the HIV Epidemic Through Point-of-Care Technologies (EHPOC) randomized clinical trial conducted from August 18, 2021, to February 2, 2023, have been previously reported^12^ and are given in the trial protocol provided in Supplement 1. Briefly, eligible adults at least 18 years of age were enrolled after obtaining written informed consent. Participants included PWH and individuals with risk factors for HIV acquisition who were not receiving daily oral or regular injectable ART or PrEP. Risk factors included not identifying as heterosexual; receiving sexually transmitted infection, HIV, or hepatitis C virus testing or treatment; and reporting multiple sexual partners, condomless sex with more than 1 partner, or injection drug use. We reported outcomes from participants randomized to receive or not receive a traditional laboratory-based HIV VL assay during the enrollment visit. This report followed the Consolidated Standards of Reporting Trials (CONSORT) guideline for randomized clinical trials.^13^ This study was reviewed and approved by the Johns Hopkins School of Medicine institutional review board. Prior to study enrollment and any study procedures, written informed consent was obtained from all participants. Study procedures were performed in accordance with the relevant regulations of the Johns Hopkins University and the Declaration of Helsinki.^14^

### Participant Recruitment

Participants were recruited from 3 sources, an emergency department (ED) in Baltimore, Maryland, a social media advertising campaign (posts on Facebook and Instagram), and other sources, which included passive recruitment from an infectious diseases clinic in Baltimore, Maryland, using study flyers; an online sexually transmitted infection testing platform website^15^; and other traditional advertising, such as posters in public places. In the ED, patients who met any eligibility criteria were identified via medical record review by research coordinators (M.H.B., A.B., T.B., T.J.S., N.J., and B.R.) and, if consent was obtained, enrolled on site. Other potential participants underwent telephone eligibility screening and, if eligible, were invited to the clinical research unit at Johns Hopkins Hospital. Using SealedEnvelope^16^ online software, participants were randomly assigned 1:1 to the control or intervention group. Control group participants received standard of care (SOC) laboratory HIV screening tests; intervention group participants received SOC tests plus an HIV VL assay.

All participants completed a self-administered baseline sociodemographic and behavioral questionnaire via the Research Electronic Data Capture platform on a tablet device. Race was self-classified by participants. Race and sexual orientation were assessed and reported in the study to investigate characteristics of groups disproportionality affected by HIV and differences in PrEP access. For the analysis, due to small numbers in each category, an other race category was created in addition to Black or African American and White categories. The other category included American Indian or Alaska Native, Asian, multiracial, Native Hawaiian or Other Pacific Islander, and declined to answer.

At the enrollment visit, participants nominated a health care professional or clinic that they wished to be referred to for LTC; for participants without a preference, a choice of health care professional was supplied. Within 24 hours of laboratory results being available, research coordinators (M.H.B., A.B., T.B., T.J.S., N.J., and B.R.) communicated test results by telephone to participants and their designated health care professional (by telephone or Health Insurance Portability and Accountability Act–compliant messaging).

The final 12-week study visit (up to 14 days before and 21 days after the exact date) was conducted in person for all participants at the clinical research unit. LTC was defined as having at least 1 interaction (a telehealth or in-person conversation about HIV PrEP or ART) with a health care professional during the 12-week period after enrollment, independent of PrEP or ART initiation or reinitiation.

### Laboratory Tests

We performed SOC HIV diagnostic tests on all participants’ plasma samples using the Elecsys HIV combi PT (Roche Diagnostics) assay, and if this assay was reactive, we used the Geenius HIV-1/2 Supplemental Assay (Bio-Rad Laboratories). In the event of discordant screening test results, the cobas HIV-1 assay (Roche Molecular Systems) for quantification of HIV-1 RNA was performed. The cobas HIV-1 assay was also performed for all intervention group participants on the initial blood draw. Additionally, ED clinicians could order HIV VL testing for clinical reasons, regardless of study arm. An HIV VL lower than 20 copies/mL was considered undetectable. The HIV VL assay was conducted in batches each weekday.

### Statistical Analysis

Statistical analysis was performed using Stata SE, version 18.0 (StataCorp LLC). We assessed LTC as the primary outcome. Secondary outcomes included time to LTC by study group (control vs intervention) and stratified by HIV status. The independent variable of interest was an HIV VL test result, and the dependent variable was LTC for ART or PrEP.

Descriptive analyses used frequencies and proportions for categorical variables and median (IQR) values for continuous variables. Cox proportional hazards regression was used to estimate hazard ratios (HRs) and 95% CIs to determine the effect of having an HIV VL test result on LTC during 12 weeks of follow-up. We assessed the proportional hazards assumption for the Cox proportional hazards model using Schoenfeld residuals. The global test showed no evidence of assumption violation (χ^2^ = 0.67, *P* = .41).

Kaplan-Meier curves were constructed to illustrate the cumulative probability of LTC over time. These curves were stratified by randomization group and various participant characteristics to assess differences in LTC rates. We conducted log-rank tests to compare linkage rates between groups.

In a post hoc analysis, we examined LTC and time to LTC for individuals with or without HIV separately. All analyses were conducted using the intention-to-treat population. A 2-sided value of *P* < .05 was considered statistically significant for all relevant analyses.

## Results

### Participant Characteristics

**Figure 1.**
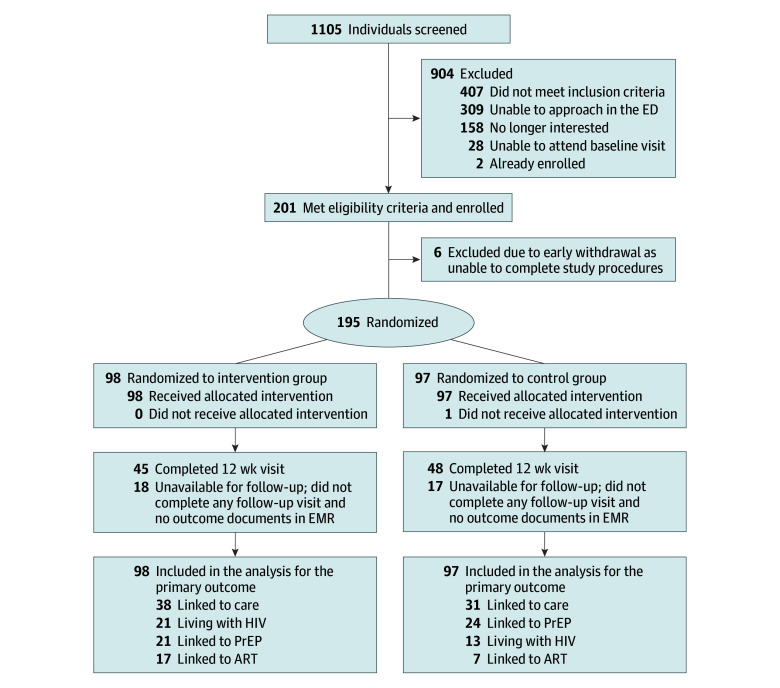
Flow Diagram of Participants Criteria for enrollment included living with HIV with unsuppressed viral load, being nonadherent to antiretroviral therapy (ART), needing reengagement to care, being at risk for HIV and not taking daily preexposure prophylaxis (PrEP), and meeting all other study inclusion criteria. One participant randomized to the control group received the intervention (HIV viral load test) ordered by the emergency department (ED) clinician. EMR indicates electronic medical record.

The median (IQR) age of participants was 36 (27-47) years, 76 (39.0)% were female, 119 (61.0%) were male, 112 (57.4%) were Black or African American, 51 (26.2%) were White, and 32 (16.4%) were other race and ethnicity. Between August 18, 2021, to February 2, 2023, 1105 potential participants were screened, and 195 (17.6%) were enrolled and individually randomized to either the control group (SOC HIV testing) or intervention group (SOC HIV testing and VL testing) (Figure 1). Of them, 97 participants were randomized to the control group and 98 to the intervention group. At enrollment, all participants with HIV (34 of 195 [17.4%]) except 1 were aware of their HIV status; of them, 16 (47.1%) had a detectable HIV VL (7 of 16 [43.8%] <200 copies/mL and 9 of 16 [56.3%] ≥200 copies/mL). Table 1 describes baseline characteristics of all study participants and by randomization group; groups were well balanced except that there were more females and fewer participants who did not have health insurance in the control group. The median (IQR) turnaround time was 6.4 (3.5-16.7) hours to laboratory-based HIV Ag/Ab assay results and 26.0 (24.0-28.7) hours for VL test results.

**Table 1.** Baseline Characteristics of Participants in the EHPOC Trial by Study Group

Characteristic	Participants, No. (%)^a^		
	Total	Control	Intervention
Total No.	195 (100)	97 (50.0)	98 (50.0)
Age, median (IQR), y	36 (27-47)	36 (26-46)	37 (28-49)
18-24	36 (18.5)	18 (18.5)	18 (18.0)
25-40	84 (43.1)	45 (46.5)	39 (40.0)
>40	75 (38.5)	34 (35.0)	41 (42.0)
Sex			
Female	76 (39.0)	46 (47.4)	30 (30.6)
Male	119 (61.0)	51 (52.6)	68 (69.4)
Race			
Black or African American	112 (57.4)	54 (55.7)	58 (59.2)
White	51 (26.2)	28 (28.9)	23 (23.5)
Other^b^	32 (16.4)	15 (15.5)	17 (17.3)
Sexual orientation			
Heterosexual	125 (64.1)	62 (63.9)	63 (64.3)
Not heterosexual	70 (35.9)	35 (36.1)	35 (35.7)
Educational attainment			
<High school or high school graduation	104 (53.3)	48 (49.5)	56 (57.1)
Some college up to graduate school	91 (46.7)	49 (50.5)	42 (42.9)
Participant source			
Emergency department	123 (63.1)	58 (59.8)	65 (66.3)
Other^c^	72 (36.9)	39 (40.2)	33 (33.7)
Condom use			
Not sexually active	56 (28.7)	31 (32.0)	25 (25.5)
Sex with a condom	37 (19.0)	20 (20.6)	17 (17.3)
Sex without a condom	102 (52.3)	46 (47.4)	56 (57.1)
Drug use^d^			
No	157 (80.5)	76 (78.4)	81 (82.7)
Yes	38 (19.5)	21 (21.6)	17 (17.3)
Current health insurance			
No	21 (10.8)	5 (5.2)	16 (16.3)
Yes	174 (89.2)	92 (94.8)	82 (83.7)
Current regular health care professional			
No	39 (20.0)	18 (18.6)	21 (21.4)
Yes	156 (80.0)	79 (81.4)	77 (78.6)
HIV status			
Negative	161 (82.6)	84 (86.6)	77 (78.6)
Positive	34 (17.4)	13 (13.4)	21 (21.4)

Abbreviation: EHPOC, Ending the HIV Epidemic Through Point-of-Care Technologies.

^a^ Percentages may not equal 100 due to rounding.

^b^ Other includes American Indian or Alaska Native, Asian, multiracial, Native Hawaiian or Other Pacific Islander, and declined to answer.

^c^ Other includes in-house social media, an online sexually transmitted infection testing website, and an infectious diseases clinic.

^d^ Includes cocaine, methamphetamine, and injection drug use.

Most participants (123 [63.1%]) were recruited from the ED, with smaller proportions from social media (52 [26.7%]) and other sources (20 [10.3%]). Participants recruited via social media compared with the ED were more likely to be younger than 25 years (20 [38.5%] vs 14 [11.4%]), male (33 [63.5%] vs 67 [54.5%]), White (18 [34.6%] vs 29 [23.6%]), and not heterosexual (33 [63.5%] vs 28 [22.8%]), with college-level or higher educational attainment (41 [78.8%] vs 39 [31.7%]), and were less likely to report substance use (1 [1.9%] vs 36 [29.3%]) and to be PWH (1 [1.9%] vs 26 [21.1%]).

### Linkage to Care

Of 93 (47.7%) participants who attended the 12-week follow-up visit, 69 (35.4%) received LTC by week 12. Among those participants, 38 of 69 (55.1%) and 31 of 69 (44.9%) were in the intervention and control groups, respectively; there was no significant difference in LTC (primary outcome) between intervention and control participants (HR, 1.28 [95% CI, 0.80-2.05]; *P* = .31) (Table 2). Table 2 also describes subgroup analyses of baseline characteristics by randomization group at 12 weeks after enrollment. LTC was significantly higher among participants who were not sexually active in the intervention group compared with the control group (HR, 2.56 [95% CI, 1.06-6.19]; *P* = .04).

**Table 2.** Trial Outcome and Subgroup Analyses

Outcome and analyses	No. of participants linked to care/total No. linked to care in group (%)		LTC HR (95% CI)	*P* value
	Control (n = 97)	Intervention (n = 98)		
**Primary outcome**				
Linked to care	31/97 (32.0)	38/98 (38.8)	1.28 (0.80-2.05)	.31
**Subgroup analyses**				
Age, y				
18-24	6/31 (19.3)	5/38 (13.1)	0.77 (0.23-2.52)	.66
25-40	14/31 (45.2)	18/38 (47.4)	1.62 (0.80-3.26)	.18
>40	11/31 (35.5)	15/38 (39.5)	1.21 (0.56-2.64)	.62
Sex				
Female	14/31 (45.2)	12/38 (31.6)	1.47 (0.68-3.18)	.33
Male	17/31 (54.8)	26/38 (68.4)	1.19 (0.65-2.20)	.57
Race				
Black or African American	16/31 (51.6)	22/38 (57.9)	1.30 (0.74-2.67)	.30
White	8/31 (25.8)	7/38 (18.4)	1.08 (0.39-3.00)	.87
Other^a^	7/31 (22.6)	9/38 (23.7)	1.05 (0.39-2.82)	.92
Sexual orientation				
Heterosexual	15/31 (48.4)	18/38 (47.4)	1.20 (0.61-2.38)	.59
Not heterosexual	16/31 (51.6)	20/38 (52.6)	1.43 (0.74-2.76)	.29
Educational level				
<High school or high school graduation	14/31 (45.2)	17/38 (44.7)	1.07 (0.53-2.16)	.86
Some college up to graduate school	17/31 (54.8)	21/38 (55.3)	1.59 (0.84-3.01)	.16
Participant source				
Emergency department	9/31 (29.0)	20/38 (52.6)	2.07 (0.94-4.56)	.07
Other^b^	22/31 (71.0)	18/38 (47.4)	1.05 (0.56-1.96)	.88
Condom use				
Not sexually active	8/31 (25.8)	13/38 (34.2)	2.56 (1.06-6.19)	.04
Sex with a condom	6/31 (19.4)	6/38 (15.8)	1.19 (0.38-3.68)	.77
Sex without a condom	17/31 (54.8)	19/38 (50.0)	0.92 (0.48-1.76)	.79
Drug use^c^				
No	27/31 (87.1)	30/38 (79.0)	1.04 (0.62-1.76)	.87
Yes	4/31 (12.9)	8/38 (21.0)	3.17 (0.95-10.57)	.06
Current health insurance				
No	2/31 (6.5)	6/38 (15.8)	0.85 (0.17-4.25)	.85
Yes	29/31 (93.5)	32/38 (84.2)	1.32 (0.80-2.18)	.28
Current regular health care professional				
No	3/31 (9.7)	5/38 (13.2)	1.54 (0.37-6.45)	.55
Yes	28/31 (90.3)	33/38 (86.8)	1.26 (0.76-2.09)	.36
HIV status				
Negative	24/31 (77.4)	21/38 (55.3)	0.94 (0.52-1.69)	.83
Positive	7/31 (22.6)	17/38 (44.7)	2.33 (0.95-5.68)	.06

Abbreviations: HR, hazard ratio; LTC, linkage to care.

^a^ Other includes American Indian or Alaska Native, Asian, multiracial, Native Hawaiian or Other Pacific Islander, and declined to answer.

^b^ Other includes in-house social media, an online sexually transmitted infection testing website, and infectious diseases clinic.

^c^ Includes cocaine, methamphetamine, and injection drug use.

### Linkage to ART Among PWH, and HIV PrEP Among Participants Without HIV

For 34 PWH, the intervention was not associated with LTC (HR, 2.33 [95% CI, 0.95-5.68]; *P* = .06). LTC was significantly more likely among PWH with more than a high school education (eTable 1 in Supplement 2). In 161 PrEP-eligible participants (83.6%), the intervention was not associated with LTC (HR, 0.94 [95% CI, 0.52-1.69]; *P* = .83); participants whose sexual orientation was not heterosexual, who had more than a high school education, and who were enrolled from social media and other sources were associated with higher LTC (eTable 2 in Supplement 2).

### Time to LTC

**Figure 2.**
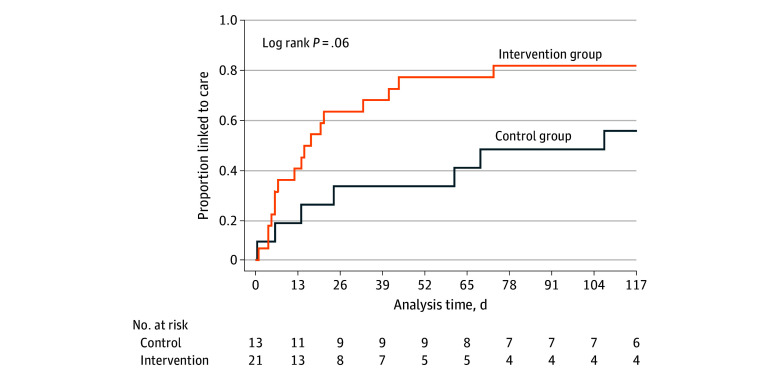
Time to Linkage to Care Kaplan-Meier curves comparing time to linkage to care between the control and intervention groups.

Overall, there was no significant difference in time to LTC between the intervention and control groups. Kaplan-Meier curve estimates comparing time to LTC for participants living with HIV in comparator groups are shown in Figure 2. In a post hoc analysis, there was a nonsignificant reduction in time to LTC among PWH who received a VL test result (intervention group) (log-rank *P* = .06). In the modified intention-to-treat analysis, 1 participant randomized to the control group who received an HIV VL test ordered by their ED clinician was excluded from the analysis. This analysis showed a significant reduction in time to linkage to ART in the intervention group (log-rank *P* = .03); by study day 50, 16 of 21 (76.2%) in the intervention group compared with 3 of 12 (25.0%) in the control group were linked to ART (eFigure in Supplement 2).

In post hoc analyses, there was no observed difference in time to PrEP linkage for the intervention group (eTable 3 in Supplement 2). The proportion linked to PrEP by the final study visit was significantly greater among participants who identified as not heterosexual (vs heterosexual), participants with more than a high school education (vs high school or less), and participants recruited from social media and other sources (vs from the ED) (eTable 3 in Supplement 2).

## Discussion

In this randomized clinical trial, providing a next-day HIV VL test result in addition to the SOC HIV Ag/Ab test result was not associated with greater LTC compared with provision of the SOC HIV Ag/Ab test result alone. More than one-third of participants pursued linkage to ART or PrEP within 12 weeks; however, more women and higher insurance coverage in the control group may have biased the results toward the null. Other studies have described lower linkage to PrEP over the same time frame—up to 24% in 2 populations at elevated risk of HIV in Chicago between 2015 and 2017.^17^ Participants recruited from an urban ED, an acute episodic care setting, had a particularly low follow-up rate, as has been previously reported; in an analysis of 3 studies in EDs, 2.3% to 19.4% of PrEP-eligible patients were linked to care.^18^ Patients who attend acute care settings may focus on acute needs, rather than longer-term preventive care, and may therefore be less likely to be linked to PrEP.^18^

There were some notable secondary outcomes in post hoc analyses. Among PWH, receipt of a VL test result led to 2-fold greater LTC compared with those who did not receive the result, although the increase was not statistically significant. Participants with established HIV in the intervention group had a significant reduction in time to LTC compared with control participants. Receipt of a VL test result had no significant effect on LTC or time to linkage in people without HIV. Relinkage to care among persons already diagnosed with HIV is essential; the CDC estimated that 80% of new HIV infections in the US were associated with PWH who were diagnosed but not in care.^19^ In 2022, 84% of PWH knew their status, 47% were retained in care, but the majority were out of care and likely not consistently virally suppressed.^20^ PWH are not a homogenous population; thus, different approaches are needed for PWH already diagnosed but not in care. In a study of EDs in San Diego, California, 85% of persons newly diagnosed with HIV in the ED were linked to care, compared with 52% of known PWH who were out of care.^21^ Other studies have shown much higher LTC in patients with acute HIV.^22^

Several interventions to reduce the time to relinkage to ART in the US have been unsuccessful.^23,24^ However, other interventions have demonstrated reduced time to relinkage but not increased retention in care.^25^ The Cooperative Re-Engagement Controlled trial demonstrated that the proportion of PWH relinked at 90 days was higher in the intervention group (active follow-up; 54.9%) compared with the control group (SOC; 42.1%).^26^ Modeling studies have demonstrated that increasing the proportion of PWH in care could have prevented 52% of new infections that occurred from 2016 to 2025.^27^ In the present trial, among participants eligible for PrEP, identifying as not heterosexual, having a higher educational attainment, and being enrolled outside of the ED were associated with higher LTC, suggesting a need to reach other groups who would benefit from PrEP.

The mechanisms underpinning these observations are obscure. Patient-reported experience measures on VL testing are limited, particularly in high-income settings. In a qualitative study among PWH in Papua New Guinea, knowledge of VL test results appeared to be motivating for continued engagement in care and adherence.^28^ In Tanzania, satisfaction with VL testing services was associated with respondents following medical advice and involvement in decision-making.^29^ Kenyan patients and their caregivers indicated that VL monitoring, in tandem with adherence counseling, encouraged ART adherence by providing an active form of feedback on how adherence was impacting their health.^30^ Similarly, among pregnant women in Uganda, POC HIV VL testing motivated ART adherence.^31^ Other studies in low- and middle-income countries^32^ and high-income settings^33,34^ have described an association between patients’ knowledge of their VL test result and positive attitudes toward treatment or medication adherence. Taken together, these studies suggest that providing HIV VL test results have a positive effect on health behaviors among PWH.

Additional US-based studies are needed to examine patient-reported experience measures, including VL literacy,^35,36,37,38^ to understand how HIV VL test results are understood by patients and whether they influence engagement in HIV care behaviors after the results are communicated. As was observed in the present trial, most HIV VL tests are conducted in centralized laboratories with lengthy turnaround times. Reviews of available and in-development HIV POC VL tests have been published previously.^39^ Studies in sub-Saharan Africa have demonstrated how POC HIV VL tests, when combined with task-shifting, can result in improved treatment outcomes compared with traditional HIV diagnostic testing alone.^40^

The turnaround times of HIV VL test results may be a critical factor in LTC success. In a Nigerian study, the timeliness of providing the HIV VL test result was associated with better outcomes among PWH taking ART; in patients receiving ART with viremia, POC VL monitoring with a wait time of 2 to 3 hours markedly reduced time to switch to second-line ART regimens from 66 days to 0 days.^9^ In Kenya, key informants unanimously acknowledged the benefits of rapid turnaround times.^30^ Our study demonstrated in a post hoc analysis that even with a turnaround time of more than 24 hours, providing a VL test result was associated with earlier engagement in HIV services among PWH. The next-day, centralized laboratory-based HIV VL test result that was available in our academic center setting is not available in all EDs or in nontraditional settings, such as outreach services; affordable, small-footprint POC VL tests could overcome some of these barriers. However, studies describing the utility of POC HIV VL tests have not been replicated in the US because an FDA-approved test of this type is not currently available.

### Limitations

Our study has several limitations. A median turnaround time of more than 24 hours necessarily precludes using the VL test results to inform same-day ART or PrEP initiation or reinitiation; therefore, opportunities for rapid LTC may have been lost. Feasibility, cost, and infrastructure barriers to broader implementation of next-day HIV VL test results limit generalizability to settings lacking onsite laboratory capacity. Qualitative data or direct patient-reported outcomes were not captured; therefore, we were unable to identify reasons for linkage or nonlinkage. The number of PWH enrolled was low, as were the proportion with a detectable VL, although most of these latter participants were recruited in the ED, which provides services to people with poor access to health care. The PWH subgroup analysis had limited statistical power, and the findings should be interpreted with appropriate caution. In addition, unavailability for follow-up was common; approximately half of enrolled participants returned for their final study visit, which may have underestimated true LTC activities and limits generalizability of study results.

## Conclusions

In this randomized clinical trial assessing the effects of HIV VL test result provision on linkage to ART or PrEP, there was no significant difference in LTC overall by control or intervention group. The literature indicates that LTC success is higher among PWH who were newly diagnosed compared with those with established infection.^21^ Overall, a laboratory-based HIV VL test result, with a mean turnaround time of 26 hours, positively influenced LTC and time to LTC in our sample of PWH in Baltimore, Maryland. Future research might investigate testing of HIV VL assays with shorter turnaround times, especially assays that can provide results during the clinical encounter. Further, more comprehensive, packaged interventions, such as rapid VL testing with immediate initiation or reinitiation of ART or PrEP, may be beneficial for HIV treatment and prevention.

## Notes

Supplement 1.
Trial Protocol

Supplement 2. **eTable 1.** Unadjusted Hazard Ratios for Linkage-to-Care Among 34 Persons With HIV **eTable 2.** Unadjusted Hazard Ratios for Linkage-to-Care Among 161 Persons Without HIV **eTable 3.** Time to Linkage: Proportion Linked-to-Care Among 161 Persons Without HIV **eFigure.** Kaplan-Meier Curve Comparing Time to Linkage to Care Between Groups

Supplement 3.
Data Sharing Statement
