# Supplementary material for: Next-Day HIV Viral Load Test Result and Linkage to Care Among Persons Living With or at Risk of HIV: A Randomized Clinical Trial
Source: JAMA Netw Open. 2025 Dec 16;8(12):e2548380. doi: 10.1001/jamanetworkopen.2025.48380 (PMC12709377; doi:10.1001/jamanetworkopen.2025.48380)
Supplement: Supplement 1. — Trial Protocol [file jamanetwopen-e2548380-s001.pdf]

JHM IRB - eForm A – Protocol

- **Use the section headings to write the JHM IRB eForm A, inserting the appropriate material in each. If a section is not applicable, leave heading in and insert N/A.**
- **When submitting JHM IRB eForm A (new or revised), enter the date submitted to the field at the top of JHM IRB eForm A.**

****

ENDING THE HIV EPIDEMIC THROUGH POINT-OF-CARE TECHNOLOGIES (EHPOC): PERFORMANCE EVALUATION OF NOVEL POC HIV TESTS IN BALTIMORE

List of abbreviations:

Ab: Antibody

Ag: Antigen

AIDS: Acquired Immunodeficiency Syndrome

ART: Anti-Retroviral Therapy

CDC: Centers for Disease Control and Prevention

CLIA: Clinical Laboratory Improvement Amendments

EHE: Ending the HIV Epidemic

HIV: Human Immunodeficiency Virus

MSM: Men who have Sex with Men

NAAT: Nucleic Acid Amplification test

POC: Point Of Care

POCT: Point Of Care Test

PEP: Post Exposure Prophylaxis

PrEP: Pre Exposure Prophylaxis

RNA: Ribonucleic Acid

STI: Sexually Transmitted Infections

SOC: Standard of Care

US: United States

VL: Viral Load

1. Abstract:

a. Provide no more than a one page research abstract briefly stating the problem, the research hypothesis, and the importance of the research.

The strategy for Ending the HIV Epidemic (EHE) includes four key strategies that together can end the HIV epidemic in the United States (US): **Diagnose**, Treat, Prevent, and Respond. Diagnosis is the gateway to all other interventions; it is the cornerstone of EHE. In 2019/20 it was estimated that more than 160,000 Americans are unaware they are living with HIV[1]. Early diagnosis coupled with rapid linkage to care is critical and can lead to improved individual and community health outcomes. Achieving this goal will require improved, more accessible, and routine HIV testing; immediately connecting people with HIV to care services; and connecting those without HIV to appropriate HIV prevention services. Maryland was ranked 6th among states and territories in adult/adolescent HIV diagnosis rates (per 100,000) in 2018, tied with Mississippi. Among people living with HIV in Maryland in 2019, the Centers for Disease Control and Prevention (CDC) estimated that 89.2% had been diagnosed, and that ~3,830 people with HIV are undiagnosed[2].

Evaluation of existing and new point-of-care (POC) HIV tests is needed to inform testing guidelines and provide updated information to HIV test providers. Characterizing the performance of POC tests can provide estimates for the window period for HIV detection (i.e., the time from HIV acquisition to the time that a diagnostic test becomes positive). The window period provides key information needed to interpret an initial positive test result and assess the risk of transmission to others. It may also help guide decisions about repeat testing and initiation of ART in those with HIV and prevention interventions, including pre-exposure prophylaxis [PrEP] and post-exposure prophylaxis [PEP]) (in those without HIV)[3].

During the window period for an HIV Antigen/Antibody (Ag/Ab) test, infected individuals may have non-reactive test results, falsely reassuring patients and providers. HIV RNA (or ‘viral load’ [VL]) assays have window periods that are approximately 10 days shorter than most HIV Ag/Ab tests, providing greater sensitivity for detection of early HIV infection[4]. Use of HIV RNA detection platforms for HIV screening facilitates earlier diagnosis and more effective implementation of ART and PrEP. This may be especially useful in settings where infection is acquired in persons using PrEP, since PrEP agents may suppress viral replication and delay antibody production.

The following **hypotheses** underpin the planned study:

A. Determining the performance characteristics of HIV POC tests will inform optimal testing strategies in different populations and settings.

B. Use of HIV RNA POC tests will improve linkage to HIV treatment and prevention services.

The implications of this CDC-sponsored research are important, since this research could improve early diagnosis of HIV, reduce the time to ART initiation, and facilitate timely and appropriate referral for prevention services. Additionally, if someone is infected while using long-acting PrEP,

or initiated PrEP while infected, the risk of resistance and side effects can be minimized; if infection is missed. These are critical issues for EHE success.

2. Objectives:

Primary: To evaluate the impact of a HIV POC viral load (molecular) test on linkage to HIV care or prevention in persons with, or at higher risk of, HIV infection being seen in acute care settings.

Secondary: A. To evaluate the performance of HIV POC serologic tests using fresh whole blood and oral fluid specimens at baseline in all participants against a reference lab HIV testing algorithm

B. To evaluate the seroconversion sensitivity of the HIV tests through serial follow-up and describe characteristics of persons in the process of seroconversion compared to those of persons with established infection.

Exploratory Aim: Evaluate the diagnostic and clinical performance of the Roche cobas® Plasma Separation Cards compared to point-of-care and nucleic acid tests in clinical settings. Remnant venous blood collected from a random subset of participants (up to N=100) may be spotted on Plasma Separation Cards, stored for a minimum of 4 hours and up to 56 days and then processed. Results will be compared against plasma HIV RNA levels to determine the sensitivity of POC NAATs at varying levels of viremia.

3. Background:

(briefly describe pre-clinical and clinical data, current experience with procedures, drug or device, and any other relevant information to justify the research)

Identification of HIV infection in the acute stage serves a public health need since those with acute infection usually have high viral loads and thus, have an increased risk of transmitting infection[5]. In this phase of infection, tests based on HIV antigen and/or antibody will often be non-reactive/negative. Lack of a timely diagnosis can increase the risk of on-going HIV transmission, since initiation of ART will be delayed[6, 7]. Studies suggest that early initiation of ART has both clinical and public health benefits[6]. Although sensitivity of HIV Ag/Ab tests appears to be improving over time, these tests are not sufficiently sensitive for detecting many early/acute infections. Assays that measure HIV RNA have traditionally been complicated, available only in centralized reference labs, and expensive to perform; although a variety of strategies to use these tests for HIV screening in clinical settings have been proposed, these tests are still not widely used at the POC. In a 2019 review of 32 manuscripts, Agutu et al. examined the performance and clinical utility of HIV VL POC tests[8]. The authors concluded that HIV VL POC assay performance was comparable to reference assays, and have potential to improve patient outcomes. Of the studies examined, only two studies (with comparator testing) used the results of the POC assays for clinical management. Studies using the Cepheid GeneXpert HIV-1 Qual POC HIV VL to assist with early infant HIV diagnosis demonstrated a sensitivity of 94.1% and specificity of 99.8% compared to the standard of care Roche CAP/CTM HIV-1 qualitative PCR assay[9].

Several new HIV tests have recently been approved by the US Food and Drug Administration (FDA) for use on unprocessed specimens (e.g., fingerstick whole blood and/or oral fluid) at POC, or their approval is imminent. In a Peruvian study, the Chembio HIV-syphilis DPP test with an electronic reader demonstrated excellent sensitivity and specificity for HIV antibody detection (100.0% [97.6–100.0] and 98.7% [96.6–99.6], respectively). The detection of *Treponema pallidum* (TP) antibodies had slightly lower sensitivity (compared to HIV) with high specificity, (94.7% [89.8–97.7] and 99.7% [98.2–100.0], respectively)[10]. Syphilis can pre-date HIV infection in high risk individuals, having a dual test may be useful in identifying individuals with syphilis most likely to benefit from HIV PrEP[11-13].

Evaluation of new HIV testing technologies in high-risk populations will require evaluation of the sensitivity of these tests during the earliest stages of infection, including the acute stage, and comparing their performance against other rapid tests and the gold-standard reference lab standard algorithm. This information will help identify persons with previously undiagnosed infection, those in care who may not be taking their antiretroviral therapy, and those at high risk who may benefit from pre-exposure prophylaxis and using the results to link participants to appropriate treatment or prevention services.

4. Prior research:

In the past, the CDC Special Studies and Diagnostics team in the Behavioral and Clinical Surveillance Branch in the Division of HIV/AIDS has been involved in studies that evaluated the performance of HIV tests approved for use in the US, including rapid HIV tests intended for use at POC and laboratory-based HIV testing technologies[14]. A key finding was that OraQuick test using oral fluid samples detected fewer infections than the comparator tests[15]. Additionally, in a study of testing preferences among men who have sex with men (MSM) seeking HIV testing, O'Neal et al found that MSM often opted for oral fluid testing, even though they trusted and preferred more sensitive HIV testing methods, such as HIV Nucleic Acid Amplification Tests (NAAT)[16]. These evaluations led to the development of revised guidelines for HIV testing in the US[17]. The CDC's HIV Laboratory Branch also participated in evaluation of newly-approved testing technologies for which field performance data were not yet available[18, 19]. The Laboratory Branch is also working closely with test manufacturers to develop and refine nucleic acid tests for use at POC, in both the US and resource-limited settings[20].

Justification for the current study

The CDC provides guidelines for HIV testing and diagnosis for the US, as well as programmatic technical guidance for its grantees. Detailed evaluation of the diagnostic performance of nucleic acid (molecular) tests in real-world settings will allow the CDC to determine the applicability of this technology for use in a variety of clinical and POC settings. The CDC will use data collected in this study to inform HIV testing guidelines and provide information to HIV test providers about the appropriate use of different HIV testing technologies in different settings and for different populations.

5. Study Design/procedures:

a. Study design, including the sequence and timing of study procedures (distinguish research procedures from those that are part of routine care).

Study Schema:

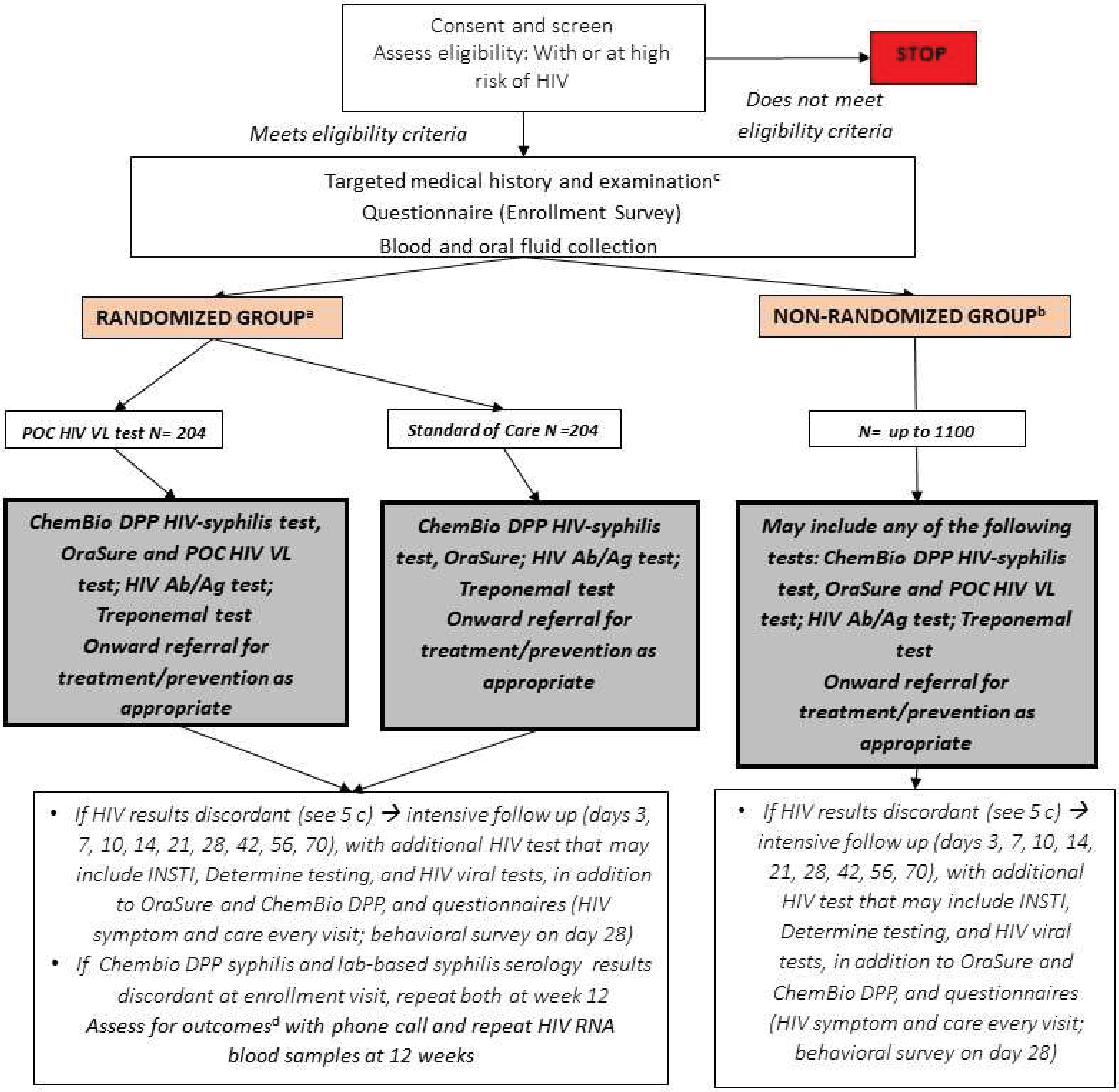

Notes:

[a]**Inclusion criteria:** MSM/TGW; IDU; Known STI or being screened for STI; High prevalence network; Living with HIV with unsuppressed HIV VL in past 6 months; Not taking daily PrEP.

[b]**Inclusion criteria:** MSM/TGW; IDU; Known STI or being screened for STI; High prevalence network; Living with HIV.

[c]**Medical exam** – abstract from clinical notes if appropriate

[d]**Outcomes**

Primary Outcome: Proportion linked either to PrEP or ART

Secondary Outcomes:

1) HIV: HIV case identification, time to linkage to PrEP or ART; proportion of positives started on ART, proportion virally suppressed at 12 weeks
2) Syphilis: linkage to syphilis treatment
3) HIV 'knowledge' – behavioral change (awareness/risk behavior change) since the time of diagnosis

Patients attending clinical services will undergo their usual clinical procedures prior to joining the study; all SOC testing will be performed as usual. Potential study participants will be asked if they are interested in taking part in the study. Those who express an interest in the study will be introduced to a member of the research team to discuss the study, assess eligibility and provide written informed consent before any study procedures take place. Those who do not agree to participate will continue with their routine clinical care.

Some potential participants might be at high risk of poor sexual health outcomes, but may not meet inclusion criteria for the randomized group because they are unable or unwilling to commit to the follow-up portion of the study or are taking daily ART or PrEP. In order to maximize equity of access to research opportunities for those described above, a non-randomized group will be made available. Those in the non-randomized group will be offered rapid and laboratory-based HIV and syphilis diagnostics, will be appropriately linked to care, and will have a telephone and/or an email follow-up interview at 4 weeks after their enrollment visit.

Inclusion:

- Aged 18 years or older
- At high risk for HIV and STIs (e.g., patients who receive STI, HIV and/or HCV testing, or treatment for STI)
- Willing to undergo phlebotomy and collection of oral fluid samples
- Willing to complete a questionnaire
- Willing to have laboratory results shared with the clinician(s) and Disease Intervention Specialists (DIS) associated with their care
- Willing to complete follow-up visits/activities
- Willing to have the research team look at the medical record to make a note of examination findings, diagnoses, and test results when required
- Willing for samples to be stored at the Johns Hopkins STD research laboratory for future research use and for samples to be transferred to the CDC for analysis and storage
- Randomized group only:
    - Those living with HIV who do not have a documented undetectable HIV viral load during the last 6 months or who do not take HIV medications every day; or, those at risk for HIV who are not taking daily PrEP

Exclusion:

- Aged <18 years
- Unwilling to undergo study procedures
- Any other reason deemed pertinent by the study team

<u>Study Procedures:</u>

At enrollment (all participants):

After informed consent, eligible participants will provide blood and oral fluid specimens which will be obtained to evaluate the performance characteristics of HIV (and syphilis) POC tests

1. Chembio dual platform for HIV and syphilis (DPP HIV-Syphilis System) – whole blood
2. OraSure OraQuick ADVANCE HIV, CLIA-waived rapid oral fluid test for HIV
3. Reverse algorithm syphilis serological testing (EIA, RPR plus titer, TPPA)
4. Collect Oral fluid (e.g. in DPP sampletainer) – process and freeze for storage in the biorepository and future lab evaluation of HIV and Syphilis at the CDC

Blood and oral fluid samples will be tested in a Clinical Laboratory Improvement Amendments (CLIA)-waived laboratory at the study sites if appropriate, otherwise testing will happen at the research laboratory. The intent is that all specimens from prospectively-enrolled participants will be tested as soon as possible after collection ('Prospective Fresh Specimens') without freezing. Specimens obtained from prospectively-enrolled participants will also be frozen and saved for future HIV test evaluation purposes.

All blood will also undergo the SOC HIV testing algorithm used at the Immunology Laboratory at Johns Hopkins Hospital (JHH). The current HIV testing protocol in this laboratory is: a laboratory-based, instrumented, Roche Elecsys HIV combi PT (an HIV-1/HIV-2 Ag/Ab immunoassay), with reflexive testing using the Geenius HIV1/2 Supplemental Assay, and 'tie-breaker' Roche Cobas Quantitative HIV-1 RNA test.

Participants will also complete a demographic, sexual behavior, and drug use questionnaire (Enrollment survey), and physical exam findings will be abstracted from the medical record if appropriate.

For the randomized group, in addition to SOC testing, participants will also be randomized to either undergo HIV VL POC testing (Cepheid GeneXpert HIV-1 Viral Load (Xpert VL; Cepheid, Sunnyvale, CA)) using blood already collected or receive SOC testing only. . Those in the non-randomized group will all undergo the Cepheid GeneXpert HIV-1 Qual test, if resources allow. If resources do not allow, the non-randomized group will undergo all standard HIV diagnostics without the HIV VL POC test. Those with confirmed HIV infection will be referred to their preferred clinician, and to the local Disease Intervention Specialist (DIS) team for linkage to care and partner services. Those with non-reactive/negative test results will be referred to their preferred clinician for consideration of HIV PrEP and prevention counselling.

Participants will receive an HIV VL result from the Johns Hopkins Molecular virology lab within 24 hours (same day) using either a validated FDA approved or a validated result from a research use only test performed in a CLIA-licensed lab. Only the results of FDA-approved testing or validated result from a research use only test performed in a CLIA-approved lab may be shared with participants. We will collect blood and transfer the sample to the Immunology Laboratory at JHH for testing. Participants will be contacted within 1 working day of results being released to clinical staff and referred to HIV prevention or treatment services. For the randomized group, in order to ensure that contact details are up-to-date and to proactively answer any participant questions we will contact participants between the 4- and 12-week visits by telephone, SMS, or email as previously agreed.

At Week 4 Visit (all participants – email and/or telephone call)

Participant: Questionnaires: HIV symptom and care; behavioral survey. Linkage to care information. Change in medication. Results of interval laboratory tests.

Study Team (abstraction from medical record): Participant symptoms, physical exam findings. Linkage to care information. Change in medication. Results of interval laboratory tests.

At Week 12 Visit (participants in randomized group only)

Participant: Questionnaires (using REDCap to be completed on site or through email as previously agreed): HIV symptom and care; behavioral survey. Provide oral fluid (e.g. in DPP sampletainer) – to be processed and frozen for storage in the biorepository and future lab evaluation of HIV and syphilis at the CDC. Linkage to care information. Change in medication. Results of interval laboratory tests.

Study Team (abstraction from medical record): Participant symptoms, physical exam findings. Linkage to care information. Change in medication. Results of interval laboratory tests.

Laboratory tests: HIV RNA testing – qual (batched) / quant (real time) – on everybody

For participants with discordant Chembio DPP Syphilis results when compared with reverse algorithm syphilis serological testing results at enrollment, both tests will be repeated.

Off-Study

Randomized group:

- After the completion of the 12-week visit, participants in the randomized group will be followed for an additional 30 days to monitor for any adverse events from the blood draw and any relevant outcomes (e.g. linkage-to-care). After this 30-day period, participants will be considered off-study and will no longer be followed.
- For participants who do not complete the 12-week visit, after the eligibility window for the visit has passed, participants in the randomized group will be followed for an additional 30 days to monitor for any relevant outcomes (e.g. linkage-to-care). After this 30-day period, participants will be considered off-study and will no longer be followed.

Non-randomized group:

- After the completion of the 4-week visit, participants in the non-randomized group will be followed for an additional 30 days to monitor for any relevant outcomes (e.g. linkage-to-care). After this 30-day period, participants will be considered off-study and will no longer be followed.
- For participants who do not complete the 4-week visit, after the eligibility window for the visit has passed, participants in the non-randomized group will be followed for an

additional 30 days to monitor for any relevant outcomes (e.g. linkage-to-care). After this 30-day period, participants will be considered off-study and will no longer be followed.

Sample storage

During the consent process participants will be asked if they consent to sample storage at Johns Hopkins. For those who provide written consent, a proportion of samples will be sent to the Point of Care Center's repository at Johns Hopkins University where they may be used to develop or test performance of STI diagnostics in the future.

b. If your study involves data/biospecimens from participants enrolled under other research studies with a written consent or under a waiver of consent, please list the IRB application numbers for those studies. Please note: Certificate of Confidentiality (CoC) protections applied to the data in source studies funded by NIH or CDC will extend to this new study if the funding was active in 2016. If this situation applies, Section 36, question 6 in the application will need to be answered "Yes" and "Hopkins Faculty" should be selected in question 7. No other documents are required.

There are no plans to enroll participants enrolled in other research studies.

c. Study duration and number of study visits required of research participants.

The study will recruit participants over a 36-month period. Each participant will be enrolled for up to 12 weeks after the Day 0 visit.

For all participants, the following visits are planned

Day 0 – enrollment

Week 4 post-enrollment (window: minus 7 days to plus 14 days)

Week 12 post-enrollment, for the randomized group only (window: minus 14 days to plus 21 days)

Persons whose initial POC test results are discordant with laboratory-based HIV test results will be offered additional study follow-up visits to evaluate the seroconversion sensitivity of the HIV tests through serial follow-up.

Discordance is defined as:

- OraQuick result is different than DPP HIV result
- OraQuick or DPP HIV are negative and lab algorithm indicates positive
- OraQuick negative and Cobas quant/Cepheid GeneXpert HIV POC VL positive
- DPP HIV negative and Cobas quant/Cepheid GeneXpert HIV POC VL positive
- Any other permutation deemed relevant by the research team

This will include people with both newly diagnosed/acute HIV infection, and persons who had at least one false reactive/positive test result. These individuals will also be linked to routine clinical services for clinical care. The participants with discordant results will be followed by the study team until one of the following events occurs:

1) They test reactive/positive for HIV for all tests being evaluated,
2) They have two consecutive study visits with non-reactive/negative results for all tests (indicating tests that had false-reactive/positive results), or
3) They complete follow up out to 70 days after the initial visit (Day 0).

The follow-up schedule will include return visits at 3, 7, 10, 14, 21, 28, 42, 56, and 70 days after the initial visit (i.e., the day of Study participation or Day 0). At each follow-up visit, participants will provide blood (via venipuncture) and oral fluid specimens for storage and testing with the HIV POC tests under evaluation (e.g., OraQuick, DPP HIV/syphilis); other laboratory HIV diagnostics where applicable and HIV RNA Quant and HIV RNA Qual as well as complete a HIV symptom and care questionnaire. Linkage to care information. Change in medication. Results of interval laboratory tests.

Day 0 – enrollment

Day 3 (window minus 1 day to plus 2 days)

Day 7 (window minus 1 day to plus 2 days)

Day 10 (window minus 1 day to plus 2 days)

Day 14 (window minus 1 day to plus 3 days)

Day 21 (window minus 3 day to plus 3 days)

Day 28 (window minus 3 day to plus 7 days)

Day 42 (window minus 7 day to plus 7 days)

Day 56 (window minus 7 day to plus 7 days)

Day 70 (window minus 7 day to plus 14 days)

d. <u>Blinding, including justification for blinding or not blinding the trial, if applicable.</u>

For the randomized group, participants will be randomized to receive either a POC HIV VL test or usual clinical care. It will not be possible to blind participants or the research team to this intervention. The participant and their clinician will be informed of the POC HIV VL result.

e. <u>Justification of why participants will not receive routine care or will have current therapy stopped.</u>

All participants will receive routine clinical care. The study will provide additional assessments to routine care and will not replace any element of routine care. There is no implication for changes in participant's current therapy.

f. Justification for inclusion of a placebo or non-treatment group.

There is no placebo group. Treatment is not a component of this study. For the randomized group, participants will be randomized to receive an additional HIV POC VL test that is not currently used in clinical practice in Baltimore to diagnose HIV infection and to guide referral for rapid ART initiation or HIV PrEP. There is sufficient equipoise to justify use of HIV POC VL testing.

g. Definition of treatment failure or participant removal criteria.

Treatment failure is not a component of this study.

Participants who provide consent and are enrolled, but who do not provide biological samples will be classified as early withdrawals.

Voluntary Withdrawal by a Participant: Participants may withdraw voluntarily from study participation at any time. Participants who withdraw will continue their care with the appropriate clinical service for evaluation and care.

h. Description of what happens to participants receiving therapy when study ends or if a participant's participation in the study ends prematurely.

NA. Treatment is not a component of this study.

i. If biological materials are involved, please describe all the experimental procedures and analyses in which they will be used.

Blood samples:

Blood:

- POC HIV and syphilis testing (DPP HIV-Syphilis System) – whole blood
- Plasma: POC HIV VL testing (Cepheid GeneXpert or comparable platform)
- Plasma: Reference laboratory-based HIV diagnostic (Roche Elecsys with reflex to Geenius and Roche cobas quantitative HIV-1 RNA tie-breaker) and HIV RNA testing (Roche cobas qualitative and quantitative test) as appropriate. Plasma remaining after HIV diagnostic testing will be retrieved by study staff and used for IRB-approved testing, and biorepository storage. All Roche cobas qualitative tests will be run retrospectively on frozen plasma with batched testing.
- Remnant blood collected from a random subset of participants (up to N=100) may be spotted on Plasma Separation Cards, stored and then processed. Results will be compared against SOC plasma HIV RNA viral load tests to determine the sensitivity of POC viral loads tests at varying levels of viremia.
- Serum: reference lab-based testing for treponemal antibody testing, and, if positive, RPR titer. Serum remaining after treponemal antibody testing will be retrieved by study staff and stored in the research laboratory for IRB-approved testing, and biorepository storage.

- For storage for testing with future HIV or syphilis assays

Oral fluid:

- OraQuick HIV antibody testing
- For storage for testing with future HIV assays (e.g., using the Chembio DPP sampletainer)

Randomization:

For participants in the randomized group, all eligible consented participants will be randomized to receive one of two testing arms using a computerized randomization system in the ratio of 1:1. Participants will be randomized to receive either POC HIV VL plus SOC testing or SOC testing only. Randomization will be performed by research staff after informed consent and verification of all eligibility criteria have been performed.

Early Withdrawals:

Participants who have provided consent and are enrolled, but who do not complete all study procedures at the time of enrollment, will be removed from the study and classified as early withdrawals. Participants may also be removed from the study early for failing to follow instructions, if continuation is deemed potentially harmful, or for any other reason at the discretion of the study principal investigator.

6. Study sites:

The following study sites are planned for the conduct of this study.

1. Johns Hopkins Medicine Emergency Department (JHMED)
2. The John G. Bartlett Specialty Practice (JGBSP), Johns Hopkins Hospital
3. The Baltimore City Health Department (BCHD) Health and Wellness Center, Sexual Health Clinics

These sites have been selected to provide optimal settings for patient interaction, and to provide access to patients at high risk of HIV infection.

Potential participants may be seen at any of the sites mentioned below or the Clinical Research Units (CRU) at the Institute for Clinical and Translational Research (ICTR).

Johns Hopkins Hospital Emergency Department (JHHED) is located in East Baltimore, Maryland. (Drs. Rothman and Hsieh). The mission of JHHED is to provide a patient-centered care experience by world-class emergency health care providers. The JHH ED serves a predominantly socioeconomically-disadvantaged population in the metropolitan area with 60% from Baltimore City, one of the localities hardest hit by the HIV epidemic and 33% from Baltimore County. JHH adult ED patient population is comprised of 15% previous or current injection drug users, with a high prevalence of sexually transmitted infections (STIs) [21-23] and 6% HIV seroprevalence[24]. In FY 2022, the JHH ED had approximately 60,000 adult patient visits. Of these, approximately 65% of the patients were Black or African American and 5% had previously-diagnosed HIV

infection [24]. The JHH ED has been a leader in ED-based HIV screening and linkage to care (LTC) programs since 2005 and currently has a robust testing and LTC program for both HIV and hepatitis C virus (HCV), operating since 2015. Past research includes evaluation of the feasibility and validation of new POC technologies.

Johns Hopkins Bayview Medical Center Emergency Department (JHBMC ED) is located in East Baltimore, Maryland (Drs. Rothman and Hsieh). The mission of the JHBMC ED is to provide a patient-centered care experience by world-class emergency health care providers. The JHBMC ED, located 3.5 miles southeast of the JHHED near the city–county border, serves non-inner-city communities with diverse, socioeconomically-disadvantaged populations in the metropolitan area. The JHBMC ED patient population is comprised of 8% current injection drug users, with a high prevalence (~14%) of sexually transmitted infections (STIs) in those aged 18-31 years. In FY 2022, the JHBMC ED had approximately 51,500 adult patient visits. Of these, approximately 44% of the patients were White or Caucasian, 33% Black or African American, and 15% Latinx. The JHBMC ED implemented an ED-based rapid POC HIV screening and linkage to care (LTC) program from 2008 to 2011, and since 2015 has operated a 4th generation venipuncture whole blood testing and LTC program for HIV. The newly diagnosed HIV infection rate from the testing program is 0.2%.

John G. Bartlett Specialty Practice (JGBSP) is part of the Johns Hopkins Medical Institutions (JHMI), a non-profit academic medical center located in East Baltimore, Maryland. Faculty from the Division of Infectious Diseases (ID) (Drs. Manabe and Hamill) in the Department of Medicine run the program; the mission is to provide outstanding, evidence-based, clinical infectious disease care, accessible to all. The population served by this practice is similar to the patient population of the JHH ED. This practice is a trusted local provider of HIV treatment and prevention services and provides care to more than 3,000 people living with HIV as well as patients with viral hepatitis, general ID, and transplant ID.

This site has experience with on-site recruitment of clinician-collected and self-collected swabs for POC STI diagnostic testing.

The Baltimore City Health Department (BCHD) Sexual Health and Wellness Clinics currently serve approximately 16,000 patients per year for STI testing, treatment and care. It operates two clinics one in East Baltimore (Fayette Street) and one clinic in West Baltimore (Druid). These areas have high rates of STIs, HIV, opioid use, and social deprivation. The clinics are each open 5 days per week and provide care for STI, HIV, HCV and PrEP. The clinical services are headed by Clinical Chiefs in sexually transmitted disease (STD) (Dr. Hamill). The Johns Hopkins University School of Medicine (JHU SOM) has a greater than 30-year collaborative relationship with the HIV/STD Program at the BCHD.

Routine opt-out serologic testing for HIV is provided. In many cases, testing is offered more frequently for high-risk patients, including patients on or eligible for PrEP.

BCHD's two sexual health and wellness clinics also play an important role for community members who may not otherwise have access to health-care services, including those who are uninsured/underinsured and at high risk for HIV.

We will also recruit potential participants through the use of flyers and by direct provider referral from Johns Hopkins Community Physicians (JHCP). JHCP will partner with the study team to allow communication between the study team, clinicians, and potential participants through the use of flyers and study information. JHCP providers may also directly refer their patients to the study team if they (the patients) express an interest in learning more about the study. JHCP serves patients in more than 40 locations throughout Maryland and Washington, D.C. JHCP also provides access to a network of Johns Hopkins specialists. Their vision is to assist patients to achieve their best health by bringing Johns Hopkins Medicine to their communities. Associated with the Johns Hopkins Medical Institutions (JHMI), JHCP serves as a trusted source for excellent patient care in the communities it serves.

Non-clinic-based recruitment:

To maximize recruitment efforts and facilitate access to research to the broadest possible community, we will invite members of the public to consider joining the study. We will target key populations, including PrEP users, MSM, and transgender people through outreach to trusted community-based organizations (CBOs). Information will be made available through social media and more traditional methods, such as posters and flyers. Individuals who contact the study team expressing an interest in the study will be invited to the ICTR CRUs.

Prior to study enrollment, the study team will confirm that people who are recruited via non-clinic venues have a medical provider who can be contacted with the results of positive study laboratory results. If a participant does not have a provider, the study team will confirm that they are willing to be referred to an appropriate provider for care and treatment.

Venue for follow up visits and sample collection

Study participants who are recruited from the non-clinical sites described above will be asked to come to the ICTR-CRUs. Similarly, for those with discordant HIV results (recruited from any location) will attend the CRU, Bartlett Clinic, and BCHD for their follow up visits.

7. Drugs/ Substances/ Devices:

a. The rationale for choosing the drug and dose or for choosing the device to be used.

In this study, we will be testing performance of point of care (POC) devices, Chembio DPP HIV-Syphilis and OraQuick Advance HIV test, for rapid detection of HIV and Syphilis antibodies. Participants in the intervention (POC) arm will also receive nucleic acid amplification test for HIV viral load quantification using Roche Cobas HIV-1 Realtime PCR test and/or Cepheid GeneXpert HIV-1 viral load test.

We acknowledge in-kind contribution (non-monetary support), as Roche Diagnostics and Cepheid have agreed to provide test kits (cartridges) for this study.

b. Justification and safety information if FDA approved drugs will be administered for non-FDA approved indications or if doses or routes of administration or participant populations are changed.

NA

c. Justification and safety information if non-FDA approved drugs without an IND will be administered.

NA

8. Study Statistics:

a. Primary outcome variable.

Proportion of participants linked either to PrEP or ART

b. Secondary outcome variables.

Syphilis: linkage to syphilis treatment and time to linkage
HIV: time to linkage to either PrEP or ART; HIV case identification; proportion of persons with HIV started on ART; proportion of persons with HIV and on ART who are virally suppressed at 12 weeks.
HIV 'knowledge' – behavioral change (awareness/risk behavior change) since the time of diagnosis.

c. Statistical plan including sample size justification and interim data analysis.

With a LTC rate of 30% for known positive ED patients who are not in care and a ballpark estimated LTC rate of 50% in STD patients, this translates into an aggregate LTC rate of 40% (assuming 50% patient enrollment in the ED and 50% in the STD clinics). For the randomized group, assuming that the aggregate LTC rate will increase from 40% to 55% after the intervention (POC VL testing), a total of 173 participants are required to enroll in each arm to have a power of 80% to detect the increase of LTC rate from 40% to 55% with a two-sided significant level of 0.05. Factoring in 15% non-response rate, we will need 204 participants in each arm, respectively.

For the non-randomized group, we will recruit up to 1100 participants for the purpose of testing the performance characteristics of rapid tests for HIV and syphilis against SOC laboratory tests, and to bank biospecimens for future evaluation of new POC assays.

We will first analyze descriptive data to determine the proportion of participants linked either to PrEP or ART and the time to linkage; the proportion HIV positive; the proportion with no prior syphilis history who needed treatment; the proportion in which the treponemal test had no added value; the proportion of patients who would still be in the ED/STD clinic at the time the test resulted (for PrEP or ART provision); the proportion of those testing HIV positive who were started on ART; the proportion of those testing positive and started on ART who were virally suppressed at 12 weeks; and the proportion of those with syphilis who are linked to syphilis treatment. This will

be followed by bivariate analysis to explore the potential association between the independent variable (POC HIV VL test) and other covariates with the outcome variable of interest. Then, we will perform multivariate regression to determine the association between having a POC HIV VL test and linkage to PrEP or ART, with adjustment of covariates and confounders.

We will perform survival analysis (Cox proportional hazard model) to model the time to linkage to PrEP or ART by study arm with adjustment of covariates and confounders. We will also evaluate the performance of the ChemBio DPP HIV test and OraSure oral-fluid assay by calculating the sensitivity, specificity, positive predictive value (PPV), and negative predictive value (NPV).

d. Early stopping rules

NA

e. Secondary Analyses

- Performance accuracy of ChemBio DPP HIV test and OraSure compared to SOC algorithm for HIV
- Seroconversion sensitivity of ChemBio DPP HIV test and OraSure compared to SOC algorithm for HIV
- Performance accuracy of the DPP syphilis test vs lab-based treponemal test;
- Proportion HIV positive
- Performance accuracy of the POC VL test vs quant and qual Roche VL
- Agreement between HIV VL using plasma derived from remnant blood samples and HIV VL tested on plasma from separation cards compared
- Proportion with no syphilis history who needed treatment;
- Proportion in which the treponemal test had no added value
- Turn-around time of the POC test to determine if the patients would have been in the ED/STI clinic at the time the test result became available for the clinician (PrEP or ART provision)

f. Data Management Plan

Collection: JHU will be responsible for collecting all data for this program. JHU project staff will enroll participants into the study. Participants will complete computer-assisted self-interviews and the data will be stored in an electronic high-security password-

protected environment. Blood and oral fluid samples will be collected and submitted for laboratory analyses. Medical record information, including laboratory results, will be abstracted and linked to the individuals study record. All study data will be deidentified before submission to CDC.

Protection: To ensure that each respondent's information is protected, a unique study ID will be created for each participant and will be the only identifier included on the data collection instruments. Paper forms, when used, will be filed by the unique ID and date of interview and stored under lock and key; information collected on paper will be entered into the appropriate data system at the clinical study site and the paper forms will be destroyed within 5 years after the study has ended. In addition to the data policy, JHU will implement security procedures for any recipient systems, and information contained therein that cover all aspects of data handling for hard copy and electronic data, including:

- Ensuring project data are secured against improper disclosure or unauthorized use of information.
- Access to information only on a need-to-know basis, when necessary, in the performance of assigned duties.
- Notify the supervisor, the Project Director, and the CDC PO, within one (1) hour, if information has either been disclosed to an unauthorized individual, used in an improper manner, or altered in an improper manner.
- Report immediately to both the Project Director and the CDC PO all contacts and inquiries concerning information from unauthorized staff and non-research team personnel.

Data sharing: CDC will receive only deidentified data and specimens. Data will be stored at CDC in secure servers with access limited to CDC staff who will conduct analyses for publications and presentations. Specimens stored in the CDC Biorepository.

A Limited Data Set (LDS) will be shared with the CDC in accordance with the funding agreement. Before transmission to the CDC, the LDS will be approved by the JHM IRB.

Once the primary outcomes papers of the project have been published, data will be available from the CDC Project Officer or the JHU PI (Dr. Hamill) by email. The requestor will provide a justification for the request and agree to include project staff from JHU and CDC on all publications or preventions.

Long-term preservation of public health data: Prior to the end of the of the cooperative agreement, JHU and CDC will determine a data disposition plan. The data disposition plan may include several actions including purging or destroying data, moving data to less expensive or more secure storage like the cloud or offline, copying files to legal hold archives, or encrypting sensitive content to protect against breaches.

9. Risks:

a. Medical risks, listing all procedures, their major and minor risks and expected frequency.

The major risks of participation in these studies are primarily related to HIV testing, but may also include risks specific to the research procedures. Participants may experience increased stress secondary to having discussions about HIV infection, risk factors for acquisition of HIV infection, discussion of past medical and sexual history and discussion of how to cope with a recent HIV diagnosis.

Participants may learn that they are HIV-infected as part of these studies. Participants with acute HIV infection may have additional stress because they may be symptomatic with HIV seroconversion. Blood draws can be uncomfortable and cause bruising.

Participants may perceive a loss of confidentiality by having records accessible to study staff, study monitors and JHU regulatory or fiscal oversight staff. Participants with a reactive point-of-care HIV test will speak with a DIS at the clinic to initiate partner services. Participants who test positive on laboratory tests after testing negative on all point-of-care tests will also receive DIS support.

b. Steps taken to minimize the risks.

Participant confidentiality will be paramount at all times. All conversations about sexual and other behaviors as well as medical history, and communicating laboratory results will take place in a private area. The procedures required for these research protocols are, for the most part, standard medical procedures that are carried out by highly qualified and experienced personnel.

Stress, anxiety, and depression (for subjects diagnosed with HIV infection as part of these studies) as a result of study participation will be minimized by periodic reassurance, careful explanation of study procedures and results, maintenance of an open, supporting attitude by all study personnel, and referral back to participants' primary providers for further evaluation and care as needed. Routine clinical tests will be performed in CAP-certified laboratories to ensure accuracy of the results. Immediate care for any medical complications due to the study procedures will be provided by the investigators within their areas of competence. Participants diagnosed with HIV as part of these studies will be referred for primary care. These studies will not provide HIV care or HIV treatment.

Study documents will be retained for a minimum of 2 years after study completion, and longer if required by the sponsor. Study records will be retained until a letter approving destruction of the records is received from sponsor; this letter will be kept with the regulatory file.

Each participating site will maintain appropriate records for this study, in compliance with ICH E6, Section 4.9, regulatory and institutional requirements for the protection of confidentiality of subjects. The site participating in this study will permit authorized representatives of the sponsor(s), and regulatory agencies to examine (and when required

by applicable law, copy) clinical records for the purposes of clinical site monitoring, quality assurance reviews, audits, and evaluation of study safety and progress.

c. Plan for reporting unanticipated problems or study deviations.
Study deviations occur when there is non-adherence to the Protocol and includes Informed Consent, enrollment, and other occurrences of non-adherence to the Protocol. Protocol deviations should be sent to the JHU IRB per the JHU IRB guidelines. Any protocol deviation that meets reporting requirements of the IRB will also be reported with the same timeliness to the Johns Hopkins IRB. All deviations from the Protocol must be addressed in the study subject source documents. The documentation should include the reason(s) for the deviation and all attempts to prevent or correct the deviation. The site must complete a Protocol Deviation Form documenting each protocol deviation. A completed copy of the Protocol Deviation Form must be maintained in the regulatory file as well as in the subject's source documents.

d. Legal risks such as the risks that would be associated with breach of confidentiality.

There are no anticipated legal risks to participants based on breach of confidentiality. Utmost care will be taken to ensure that participant data are protected and secure at all times. In addition, a Certificate of Confidentiality (CoCs) protects the privacy of research participants by prohibiting disclosure of identifiable, sensitive research information to anyone not connected to the research except when the subject consents or in a few other specific situations. National Institutes of Health (NIH) funded researchers whose institutions determine that their research involves collecting or using identifiable, sensitive information are automatically deemed to be issued a CoC through their award. Several Department of Health and Human Services (HHS) agencies (including the CDC) issue CoCs for research they fund.

Certificate of Confidentiality

Consistent with Section 301(d) of the Public Health Service Act, a Certificate of Confidentiality (CoC) applies to this research because this research is funded, conducted, or supported by CDC and the following is true:

- The activity constitutes biomedical, behavioral, clinical, or other research; and
- Individually identifiable (including coded) information or biospecimens will be obtained or used for research purposes or as defined at 45 CFR 46.102(e); and
- Biospecimens are collected as part of the research, and is there a small risk that some combination of the biospecimen and other available data sources could be used to deduce the identity of an individual; and
- The research involves information about an individual for which there is at least a very small risk, that some combination of the information, a request for the information, and other available data sources could be used to deduce the identity of an individual.

Therefore, CDC and any of its collaborators, contractors, grantees, investigators or collaborating institutions that receive “identifiable, sensitive information” as defined by subsection 301(d) of the Public Health Service Act shall not:

- Disclose or provide, in any Federal, State, or local civil, criminal, administrative, legislative, or other proceeding “identifiable, sensitive information” that was created or compiled for purposes of the research, unless such disclosure or use is made with the consent of the individual to whom the information, document, or biospecimen pertains; or
- Disclose “identifiable, sensitive information” or provide ISI to any other person not connected with the research.

Disclosure is permitted only when:

- Required by Federal, State, or local laws (e.g., as required by the Food, Drug and Cosmetic Act or required by state laws requiring the reporting of communicable diseases to State and local health departments), excluding instances of disclosure in any Federal, State, or local civil, criminal, administrative, legislative, or other proceeding;
- Made with the consent of the individual to whom the information, document, or biospecimen pertains; or
- Made for the purposes of other scientific research that is in compliance with applicable Federal regulations governing the protection of human subjects in research.

CDC and its collaborators and contractors conducting this research will establish and maintain effective internal controls (e.g., policies and procedures) that provide reasonable assurance that the research is managed in compliance with subsection 301(d) of the Public Health Service Act.

CDC will ensure: 1) that any investigator or institution not funded by CDC who receives a copy of identifiable, sensitive information protected by this Certificate, understands that it is also subject to the requirements of the Certificate; and 2) that any subrecipient that receives CDC funds to carry out part of this research involving a copy of identifiable, sensitive information protected by a Certificate understands that it is subject to subsection 301(d) of the PHS Act. Therefore, all study staff will receive training on the importance of protecting the confidentiality of human research subjects and of personal information acquired, including the collection of biological specimens.

All research subjects will be informed of the protections and the limits to protections provided by this Certificate through the informed consent process. All study staff who obtain consent from study subjects will be trained on how the Certificate protects the information collected and the limitations of the Certificate’s protections.

e. Financial risks to the participants.

There are no anticipated financial risks to the participants, there is no charge for any study-related expenditure.

10. Benefits:

a. Description of the probable benefits for the participant and for society.

The participants will probably benefit by having the opportunity for earlier diagnosis of HIV and syphilis infections, this will facilitate earlier treatment and avoidance of the sequalae of untreated STI. The participant will have the opportunity to learn more about their sexual health and will be referred for rapid start of treatment/prevention services as well as risk reduction counselling with a clinician.

Society may benefit through a) the reduction of spread of STIs in the community; b) participating in a study that may change CDC guidelines and improve HIV testing that will be available to all.

11. Payment and Remuneration:

a. Detail compensation for participants including possible total compensation, proposed bonus, and any proposed reductions or penalties for not completing the protocol.

There is no payment for participating in this study. However, participants will receive a reimbursement of $40 for each in-person study visit attended and completed. Participants that complete in-person research clinic visits will also receive a parking voucher or two MTA bus tokens if applicable.

12. Costs:

a. Detail costs of study procedure(s) or drug (s) or substance(s) to participants and identify who will pay for them.

There is no charge for any study-related expenditure. All study procedures and test will be paid for by the study.

13. Transfer of Materials:

Transfer of biospecimens from Johns Hopkins to another organization for research purposes and receipt of biospecimens from an outside organization for your research must adhere to JHU policies for material transfer (https://ventures.jhu.edu/faculty-inventors/forms-policies/) and biospecimen transfer (https://hpo.johnshopkins.edu/enterprise/policies/176/39187/policy_39187.pdf?_=0.622324232879).

Please complete this section if your research involves transfer or receipt of biospecimens.

a. Will you **receive** biospecimens from an external entity for this research?

No.

If "Yes", please confirm you will secure an MTA/research agreement from the appropriate office (JHTV/ORA) prior to transfer.

b. Will you **transfer** biospecimens to an external entity as part of this research?

Yes. We will adhere to the Johns Hopkins "Transferring Human Biospecimens to Outside Organization" policy.

https://hpo.johnshopkins.edu/enterprise/policies/176/39187/policy_39187.pdf?_=0.25523869118

(See: https://ventures.jhu.edu/technology-transfer/material-transfer-agreements/ .)

If "Yes", please address each of the following:

1) Describe the nature of the research collaboration with the external entity and the rationale for the transfer. (Include an explanation of your intellectual contribution to the design of the research study, resulting data and sharing, and participation in the planned publications.)

The Johns Hopkins team has designed the protocol in collaboration with the CDC. JHU will undertake data analysis in conjunction with the CDC. The JHU team will also take the lead on any future academic publications and collaborate with the CDC in these efforts. The CDC will store these samples in a biorepository, to allow cross validation with other samples from different populations and geographical regions.

The study team will transfer deidentified processed plasma separation cards to Roche Diagnostics for HIV testing as described in the exploratory aim (page 3). JHU will only share the study ID number assigned to each participant. Roche will share the testing results with the JHU team (HIV qual and quant). The JHU team will only share the aggregate level concordance of the results as compared to SOC testing performed at JHU.

2) Please confirm you will secure an MTA through the appropriate office (JHTV or ORA) prior to transfer.

Yes

3) If the biospecimens you intend to transfer were obtained through clinical or research procedures at Johns Hopkins and "Other" is selected in Item 4, Section 23, please submit the following items in that Section:

a. A pdf version of a completed JHTV Online "Material Transfer Agreement Request Form for Outbound Material" https://ventures.jhu.edu/technology-transfer/material-transfer-agreements/ **OR** a copy of the COEUS PD (Proposal Development Summary).

Submitted

b. A completed Biospecimen Transfer Information Sheet https://www.hopkinsmedicine.org/institutional_review_board/forms/.

Submitted

A signed and dated "De-identified Human Subject Certification" https://livejohnshopkins-my.sharepoint.com/:w:/g/personal/sdamare1_jh_edu/ETANthXrGPVBmYs-_UC59fUBu9b1An7tYUWh4GjiG2fH4Q?rtime=5kjTd-F410g

Submitted

c. Approval documents from recipient site, if applicable.

d. Copies of the consent forms associated with the IRB protocols under which the biospecimens were collected, with language appropriate to this transfer highlighted.

Submitted

The name of the specialist you are working with in ORA to complete a contract/MTA.

Mr. Stephen Fischer.

Please see the following website for more information about transferring human biospecimens to outside entities: https://www.hopkinsmedicine.org/institutional_review_board/news/announcement_transfer_human_biospecimens_outside_entities.html/ .

References:

1. **Key Strategies in the Plan** [https://www.hiv.gov/federal-response/ending-the-hiv-epidemic/key-strategies]
2. **Quick Maryland HIV Statistics** [https://phpa.health.maryland.gov/oideor/chse/pages/statistics.aspx]
3. Stekler JD, Violette LR, Clark HA, McDougal SJ, Niemann LA, Katz DA, Chavez PR, Wesolowski LG, Ethridge SF, McMahan VM *et al*: **Prospective Evaluation of HIV Testing Technologies in a Clinical Setting: Protocol for Project DETECT**. *JMIR Res Protoc* 2020, **9**(1):e16332.
4. Patel P, Bennett B, Sullivan T, Parker MM, Heffelfinger JD, Sullivan PS, Group CAS: **Rapid HIV screening: missed opportunities for HIV diagnosis and prevention**. *J Clin Virol* 2012, **54**(1):42-47.
5. Miller WC, Rosenberg NE, Rutstein SE, Powers KA: **Role of acute and early HIV infection in the sexual transmission of HIV**. *Current opinion in HIV and AIDS* 2010, **5**(4):277-282.
6. Cohen MS, Smith MK, Muessig KE, Hallett TB, Powers KA, Kashuba AD: **Antiretroviral treatment of HIV-1 prevents transmission of HIV-1: where do we go from here?** *Lancet* 2013, **382**(9903):1515-1524.
7. Cohen MS, Chen YQ, McCauley M, Gamble T, Hosseinipour MC, Kumarasamy N, Hakim JG, Kumwenda J, Grinsztejn B, Pilotto JH *et al*: **Antiretroviral Therapy for the Prevention of HIV-1 Transmission**. *N Engl J Med* 2016, **375**(9):830-839.
8. Agutu CA, Ngetsa CJ, Price MA, Rinke de Wit TF, Omosa-Manyonyi G, Sanders EJ, Graham SM: **Systematic review of the performance and clinical utility of point of care HIV-1 RNA testing for diagnosis and care**. *PLoS One* 2019, **14**(6):e0218369.
9. Opollo VS, Nikuze A, Ben-Farhat J, Anyango E, Humwa F, Oyaro B, Wanjala S, Omwoyo W, Majiwa M, Akelo V *et al*: **Field evaluation of near point of care Cepheid GeneXpert HIV-1 Qual for early infant diagnosis**. *PLoS One* 2018, **13**(12):e0209778.
10. Leon SR, Ramos LB, Vargas SK, Kojima N, Perez DG, Caceres CF, Klausner JD: **Laboratory Evaluation of a Dual-Path Platform Assay for Rapid Point-of-Care HIV and Syphilis Testing**. *J Clin Microbiol* 2016, **54**(2):492-494.
11. Tilchin C, Schumacher CM, Psoter KJ, Humes E, Muvva R, Chaulk P, Checkley W, Jennings JM: **Human Immunodeficiency Virus Diagnosis After a Syphilis, Gonorrhea, or Repeat Diagnosis Among Males Including non-Men Who Have Sex With Men: What Is the Incidence?** *Sex Transm Dis* 2019, **46**(4):271-277.
12. Girometti N, Gutierrez A, Nwokolo N, McOwan A, Whitlock G: **High HIV incidence in men who have sex with men following an early syphilis diagnosis: is there room for pre-exposure prophylaxis as a prevention strategy?** *Sex Transm Infect* 2017, **93**(5):320-322.
13. Solomon MM, Mayer KH, Glidden DV, Liu AY, McMahan VM, Guanira JV, Chariyalertsak S, Fernandez T, Grant RM, Team iS: **Syphilis predicts HIV incidence among men and transgender women who have sex with men in a preexposure prophylaxis trial**. *Clin Infect Dis* 2014, **59**(7):1020-1026.
14. Delaney KP, Branson BM, Uniyal A, Phillips S, Candal D, Owen SM, Kerndt PR: **Evaluation of the performance characteristics of 6 rapid HIV antibody tests**. *Clinical infectious diseases : an official publication of the Infectious Diseases Society of America* 2011, **52**(2):257-263.
15. Stekler JD, O'Neal JD, Lane A, Swanson F, Maenza J, Stevens CE, Coombs RW, Dragavon JA, Swenson PD, Golden MR *et al*: **Relative accuracy of serum, whole blood, and oral fluid HIV tests among Seattle men who have sex with men**. *Journal of clinical virology : the official publication of the Pan American Society for Clinical Virology* 2013, **58 Suppl 1**:e119-122.

16. O'Neal JD, Golden MR, Branson BM, Stekler JD: **HIV nucleic acid amplification testing versus rapid testing: it is worth the wait. Testing preferences of men who have sex with men**. *J Acquir Immune Defic Syndr* 2012, **60**(4):e117-120.
17. CDC: **Laboratory Testing for the Diagnosis of HIV Infection: Updated Recommendations**. In. Edited by Laboratories CfDCaPaAoPH: Centers for Disease Control and Prevention and Association of Public Health Laboratories; 2014.
18. Masciotra S, Luo W, Youngpairoj AS, Kennedy MS, Wells S, Ambrose K, Sprinkle P, Owen SM: **Performance of the Alere Determine HIV-1/2 Ag/Ab Combo Rapid Test with specimens from HIV-1 seroconverters from the US and HIV-2 infected individuals from Ivory Coast**. *Journal of clinical virology : the official publication of the Pan American Society for Clinical Virology* 2013, **58 Suppl 1**:e54-58.
19. Delaney KP ES, Wesolowski L, Owen SM, Branson BM: **Performance of the Geenius HIV-1/HIV-2 Assay in the CDC HIV testing algorithm**. In: *CROI 2015: 2015; Seattle, Washington*; 2015.
20. Stevens W, Gous N, Ford N, Scott LE: **Feasibility of HIV point-of-care tests for resource-limited settings: challenges and solutions**. *BMC medicine* 2014, **12**(1):173.
21. Mehta SD, Rothman RE, Kelen GD, Quinn TC, Zenilman JM: **Clinical aspects of diagnosis of gonorrhea and Chlamydia infection in an acute care setting**. *Clin Infect Dis* 2001, **32**(4):655-659.
22. Rogers SM, Miller WC, Turner CF, Ellen J, Zenilman J, Rothman R, Villarroel MA, Al-Tayyib A, Leone P, Gaydos C *et al*: **Concordance of chlamydia trachomatis infections within sexual partnerships**. *Sex Transm Infect* 2008, **84**(1):23-28.
23. Gaydos CA, Ako MC, Lewis M, Hsieh YH, Rothman RE, Dugas AF: **Use of a Rapid Diagnostic for Chlamydia trachomatis and Neisseria gonorrhoeae for Women in the Emergency Department Can Improve Clinical Management: Report of a Randomized Clinical Trial**. *Ann Emerg Med* 2019, **74**(1):36-44.
24. Kelen GD, Hsieh YH, Rothman RE, Patel EU, Laeyendecker OB, Marzinke MA, Clarke W, Parsons T, Manucci JL, Quinn TC: **Improvements in the continuum of HIV care in an inner-city emergency department**. *AIDS* 2016, **30**(1):113-120.
