# Supplementary material for: Next-Day HIV Viral Load Test Result and Linkage to Care Among Persons Living With or at Risk of HIV: A Randomized Clinical Trial
Source: JAMA Netw Open. 2025 Dec 16;8(12):e2548380. doi: 10.1001/jamanetworkopen.2025.48380 (PMC12709377; doi:10.1001/jamanetworkopen.2025.48380)
Supplement: Supplement 2. — eTable 1. Unadjusted Hazard Ratios for Linkage-to-Care Among 34 Persons With HIV eTable 2. Unadjusted Hazard Ratios for Linkage-to-Care Among 161 Persons Without HIV eTable 3. Time to Linkage: Proportion Linked-to-Care Among 161 Persons Without HIV eFigure. Kaplan-Meier Curve Comparing Time to Linkage to Care Between Groups [file jamanetwopen-e2548380-s002.pdf]

Supplementary Online Content

Hamill MM, Bayan MH, Boudreau A, et al. Next-day HIV viral load test result and linkage to care among persons living with or at risk of HIV: a randomized clinical trial. *JAMA Netw Open*. 2025;8(12):e2548380. doi:10.1001/jamanetworkopen.2025.48380

eTable 1. Unadjusted Hazard Ratios for Linkage-to-Care Among 34 Persons With HIV

eTable 2. Unadjusted Hazard Ratios for Linkage-to-Care Among 161 Persons Without HIV

eTable 3. Time to Linkage: Proportion Linked-to-Care Among 161 Persons Without HIV

eFigure. Kaplan-Meier Curve Comparing Time to Linkage to Care Between Groups

This supplementary material has been provided by the authors to give readers additional information about their work.

eTable 1. Unadjusted Hazard Ratios for Linkage-to-Care Among 34 Persons With HIV

	Hazard Ratio (95% CI)	*P*-value
**Study arm (Ref: control group)**		
Intervention group	2.33 (0.95 – 5.68)	.06
**Age (y) (Ref: 18-24)**		
25 – 40	1.31 (0.17 – 10.38)	.80
>40	1.39 (0.18 – 10.65)	.75
**Sex (Ref: Male)**		
Female	0.74 (0.29 – 1.87)	.53
**Race (Ref: Black or African American)**		
White	0.97 (0.23 – 4.16)	.97
Other[a]	0.76 (0.18 – 3.26)	.71
**Sexual orientation (Ref: Heterosexual)**		
Not Heterosexual	0.99 (0.44 – 2.23)	.98
**Education (Ref: Less than or high school graduation)**		
Some college up to graduate School	3.81 (1.42 – 10.19)	**.008**
**Participant Source (Ref: Emergency Department)**		
Other[b]	0.89 (0.35 – 2.25)	.81
**Condom Use (Ref: Not sexually active)**		
Sex with condom	0.46 (0.14 – 1.48)	.19
Sex without condom	1.68 (0.70 – 4.06)	.25
**Drug use[c] (Ref: No drug use)**		
Yes	0.47 (0.19 – 1.13)	.09
**Current healthcare insurance (Ref: No current healthcare insurance)**		
Yes	1.75 (0.24 – 12.99)	.58
**Current regular healthcare provider (Ref: No regular healthcare provider)**		
Yes	0.13 (0.01 – 1.15)	.07
**Roche Cobas HIV-1 RNA Result (Ref: control group – no VL testing)**		
Intervention group – undetectable VL	2.94 (0.91 – 9.48)	.07
Intervention group – detectable VL	2.15 (0.84 – 5.51)	.11
**HIV Viral Load (Ref: Control group or undetectable VL)**		
VL <200 copies/mL	1.75 (0.64 – 4.76)	.27
VL ≥200 copies/mL	1.88 (0.73 – 4.86)	.19

Abbreviations: HIV, human immunodeficiency virus; Ref, referent group; VL, viral load; y, years.
[a] Other includes American Indian or Alaska Native, Asian, Multiracial, Native Hawaiian or Other Pacific Islander, and Refuse to answer.
[b] Other includes in-house social media, an online sexually transmitted infection testing website, and infectious diseases clinic.
[c] Includes cocaine, methamphetamine, injection drug use.

© 2025 Hamill MM et al. *JAMA Network Open*.

eTable 2. Unadjusted Hazard Ratios for Linkage-to-Care Among 161 Persons Without HIV

	Hazard Ratio (95% CI)	*P*-value
**Study arm (Ref: control group)**		
Intervention group	0.94 (0.52 – 1.69)	.83
**Age (y) (Ref: 18 – 24)**		
25 – 40	1.22 (0.58 – 2.57)	.60
>40	0.75 (0.33 – 1.74)	.51
**Sex (Ref: Male)**		
Female	1.23 (0.68 – 2.21)	.50
**Race (Ref: Black or African American)**		
White	1.24 (0.61 – 2.53)	.56
Other[a]	2.68 (1.33 – 5.39)	**.006**
**Sexual orientation (Ref: Heterosexual)**		
Non-Heterosexual	2.92 (1.61 – 5.29)	**.001**
**Education (Ref: Less than or high school graduation)**		
Some college up to graduate school	2.50 (1.31 – 4.76)	**.005**
**Participant Source (Ref: Emergency Department)**		
Other[b]	5.93 (2.99 – 11.74)	**.001**
**Condom use (Ref: Not sexually active)**		
Sex with condom	1.19 (0.48 – 2.95)	.71
Sex without condom	1.14 (0.56 – 2.30)	.72
**Drug use[c] (Ref: No drug use)**		
Yes	0.64 (0.25 – 1.62)	.34
**Current healthcare insurance (Ref: No current healthcare insurance)**		
Yes	0.68 (0.30 – 1.53)	.35
**Current regular healthcare provider (Ref: No current regular healthcare provider)**		
Yes	1.80 (0.80 – 4.03)	.15

Abbreviations: HIV, human immunodeficiency virus; Ref, referent group; y, years.
[a] Other includes American Indian or Alaska Native, Asian, Multiracial, Native Hawaiian or Other Pacific Islander, and Refuse to answer.
[b] Other includes in-house social media, an online sexually transmitted infection testing website, and infectious diseases clinic.
[c] Includes cocaine, methamphetamine, injection drug use.

© 2025 Hamill MM et al. *JAMA Network Open*.

eTable 3. Time to Linkage: Proportion Linked-to-Care Among 161 Persons Without HIV

Variable	**Proportion Linked by day 50 post-enrollment**	**Linked by end of study (week 12)**	**Log-rank *P*-value**
**Study arm**			.83
Intervention	22%	28%	
Control	25%	30%	
**Sexual orientation**			**.001**
Heterosexual	15%	19%	
Not heterosexual	39%	49%	
**Education**			**.004**
Up to high school education	14%	18%	
Post high school education	31%	40%	
**Recruitment site**			**.001**
Social media and other[a]	44%	57%	
Emergency Department	10%	11%	

[a] Other includes an online sexually transmitted infection testing website and infectious diseases clinic.

© 2025 Hamill MM et al. *JAMA Network Open*.

eFigure. Kaplan-Meier Curve Comparing Time to Linkage to Care Between Groups

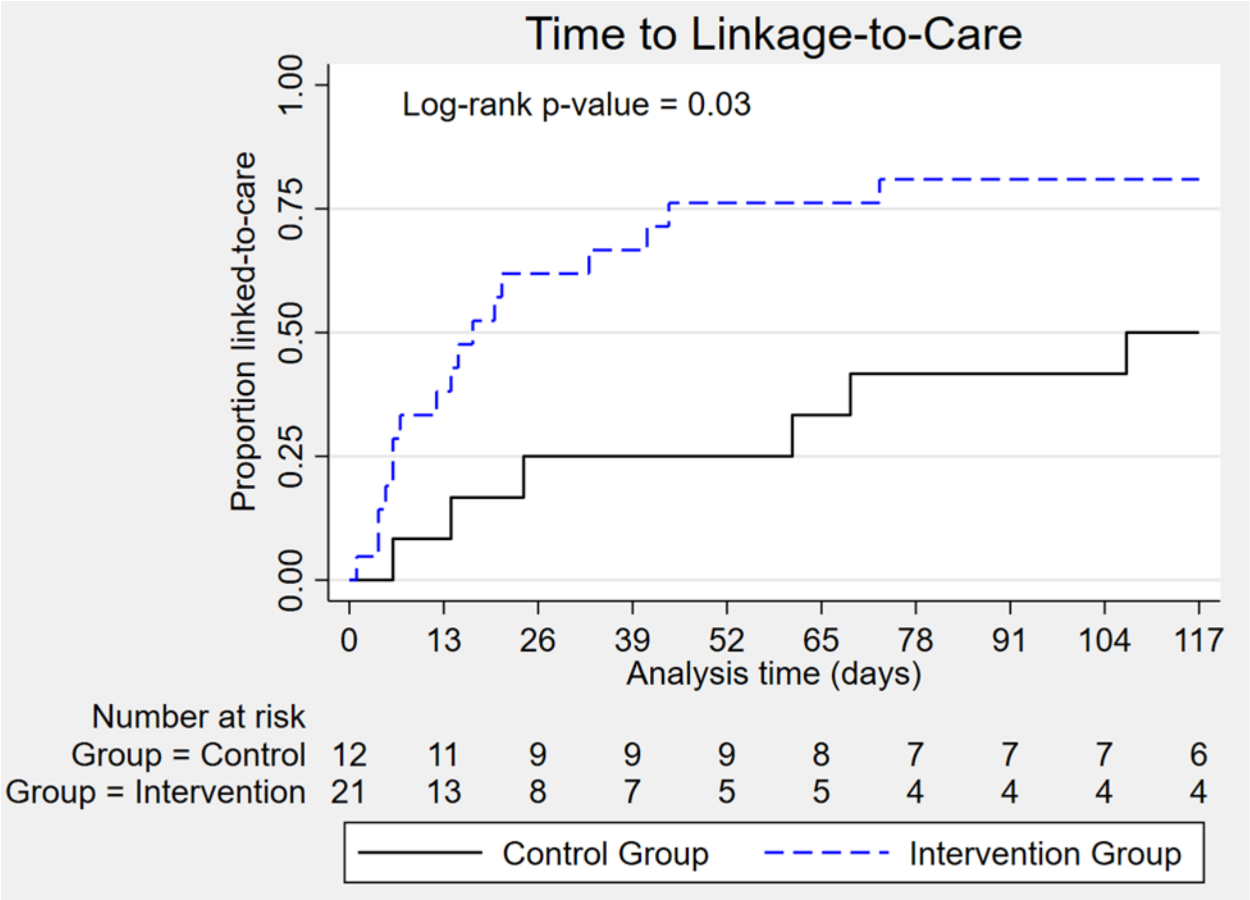

© 2025 Hamill MM et al. *JAMA Network Open*.
