# Supplementary material for: Next-Day HIV Viral Load Test Result and Linkage to Care Among Persons Living With or at Risk of HIV: A Randomized Clinical Trial
Source: JAMA Netw Open. 2025 Dec 16;8(12):e2548380. doi: 10.1001/jamanetworkopen.2025.48380 (PMC12709377; doi:10.1001/jamanetworkopen.2025.48380)
Supplement: Supplement 3. — Data Sharing Statement [file jamanetwopen-e2548380-s003.pdf]

# Data Sharing Statement

Hamill. Next-Day HIV Viral Load Test Result and Linkage to Care Among Persons Living With or at Risk of HIV. *JAMA Netw Open*. Published December 16, 2025.
doi:10.1001/jamanetworkopen.2025.48380

**Data**
**Additional Information:** NIH ClinicalTrials.gov (Identifier: NCT04793750) URL: https://clinicaltrials.gov/study/NCT04793750
**Data available:** Yes
**Data types:** Deidentified participant data
**How to access data:** mhamill6@jhu.edu
**When available:** With publication

**Supporting Documents**
**Document types:** None

**Additional Information**
**Who can access the data:** Researchers whose proposed use of the data has been approved
**Types of analyses:** Approved data plan
**Mechanisms of data availability:** Signed data access agreement
